# Violet-Blue Light Photobiological Effect on Cultured Corneal and Pigment Retinal Cells

**DOI:** 10.3390/ijms27052489

**Published:** 2026-03-08

**Authors:** Valerio Ciccone, Davide Amodeo, Gaia Papale, Alessandro Puccio, Marco Tani, Gabriele Cevenini, Lucia Morbidelli, Gabriele Messina

**Affiliations:** 1 Department of Life Sciences, University of Siena, 53100 Siena, Italy; 2 Department of Medical Biotechnology, University of Siena, 53100 Siena, Italy; 3 Department of Molecular and Developmental Medicine, University of Siena, 53100 Siena, Italy; 4 Department of Information Engineering and Mathematics, University of Siena, 53100 Siena, Italy

**Keywords:** violet-blue light, 405 nm wavelength, eye safety, pigment retinal cells, corneal cells, photobiological risk assessment, cell viability, oxidative stress, apoptosis

## Abstract

Artificial optical radiation, spanning from 100 nm to 1 mm, encompasses ultraviolet (UV) and infrared (IR) light. UV light is well known for its risks on the skin and eyes. Recently, there has been growing interest in light at 405 nm (violet-blue light, VBL) due to its antimicrobial properties and perceived safety for mammalian cells when administered in controlled amounts. This research delved into the impact of 405 nm VBL on corneal and retinal pigment epithelial cell cultures. ARPE-19 and corneal BCE C/D 1b cells were exposed to VBL for varying doses, according at different exposure times, to evaluate cell viability, oxidative stress levels and apoptotic indicators. A 3D printed prototype with 14 LEDs centred at 405 nm wavelength was used to ensure uniform distribution of light during exposure. Cell viability was assessed using the MTT assay, measurement of oxygen species (ROS) production was carried out, and Western blot analysis was employed to study catalase and SOD-1 expression and apoptotic marker activation. Exposure to 405 nm VBL for both term (3 h) and prolonged durations (9 h) led to a weak decrease in cell viability in ARPE-19 cells, whereas the effect on BCE C/D 1b cells was negligible. There was no increase in ROS production, with catalase and SOD-1 expression remaining stable, suggesting no pro-oxidative stress effects in these models. Moreover, no activation of caspase-3 and accumulation of cytochrome C were found. Based on our results, exposure to 405 nm light at regulated levels does not pose a threat to the viability of the tested cell lines and does not lead to oxidative stress and apoptosis under these conditions. These results suggest a favourable cytocompatibility profile for these specific ocular cell models, laying a foundation for further investigations into its ocular safety.

## 1. Introduction

Artificial optical radiation has a wavelength range from 100 nm to 1 mm, divided into ultraviolet (100–400 nm), visible (400–780 nm) and infrared (>780 nm) light [1]. These components, with variable effects and intensities, expose people to different photobiological risks [2]. The photobiological risk associated with a light source is assessed by studying the photochemical or thermal damage it can cause to human health, particularly to the skin and eyes [3,4].

The wavelength, intensity of optical radiation, and duration of exposure can influence photobiological risk [5]. The wavelength of incident radiation determines the type of damage that can occur, while the intensity of optical radiation determines the likelihood of damage occurring and its severity [6]. Another risk factor is the age of the exposed subject [7].

Several studies have been reported in the literature analyzing different wavelengths, exposure times and how these parameters determine photobiological risk [8,9]. One of the most extensively studied regions of the light spectrum is undoubtedly ultraviolet (UV), due to its widespread use in disinfection and therapy. UVC (100–280 nm) and UVB (280–315 nm) radiation have been associated with ocular damage such as photokeratitis and photoconjunctivitis [10,11]; it has been shown that the cornea is transparent to light in the visible spectrum and absorbs almost all UV light below 290 nm (UVC range); if exposure is uncontrolled, high levels of UV light can cause acute damage to the corneal and conjunctival epithelium, manifesting as photokeratitis and photoconjunctivitis [12]. For UVB radiation, a dose-dependent association with cortical and posterior subcapsular cataracts has been demonstrated. Ocular exposure to UVB radiation has also been associated with pterygium, climatic drop keratopathy and acute photokeratitis [13].

Acute injuries, such as erythema and burns, and chronic injuries, in particular skin cancer and accelerated skin ageing, have been demonstrated in the skin. An increased risk of developing squamous cell carcinoma, basal cell carcinoma and melanoma has been observed [14].

For wavelengths above 315 nm, from UVA to infrared radiation, skin burns have been demonstrated [15]. However, photosensitivity reactions have been shown for UVA and visible radiation, but not for infrared radiation [16,17]. Prolonged exposure to UVA radiation is responsible for early signs of photoaging and photocarcinogenic effects. UVA radiation also appears to play a key role in pigment changes that occur with age [18]. In addition, UVA appears to be involved in the development of melanoma [19]. Eye damage from UVA radiation (315–400 nm) takes the form of photochemical cataracts [20].

In recent years, there has been increasing interest in the wavelength around 405 nm [1] because it does not appear to damage mammalian cells at controlled doses [21,22]. In this context, the hypothesis of using violet-blue light (VBL) with a wavelength of 405–420 nm as a disinfection technology has been developed and has proven to be effective and safe over the years [23,24,25]. VBL, which is close to the visible spectrum, does not suffer from this limitation as its use in controlled doses is safe for humans [26]. The efficacy of VBL in inactivating various microbial species has been investigated in numerous studies, as has its safety for eukaryotic cells [27,28,29]. The results obtained justify the great interest in this wavelength, as it has also been shown to have a broad spectrum of activity against polychemoresistant bacteria and microbes prevalent in hospital environments [1,30,31].

High exposure to blue light (part of the visible light spectrum in the wavelength range 400–550 nm) causes photochemical and thermal lesions of the retina and has been implicated in the development of age-related macular degeneration (AMD) [32,33]. High short-term exposure to blue light has deleterious effects on retinal morphology [34]. In the infrared spectrum (IR), a differentiation of damage is again observed depending on the wavelength analyzed. IRA (780–1400 nm) causes cataract and retinal burn [35], IRB (1400–3000 nm) is associated with cataract and corneal burn, and IRC (>3000 nm) causes corneal burn [36]. Regulation of photobiological risk has therefore become necessary, as exposure to light sources, both artificial and non-artificial, can cause serious health damage if not adequately controlled [15]. Safety standards for optical radiation were first published in the 1990s, as it was previously believed that almost all lamps were perfectly safe [5].

The aim of this study is to evaluate the phototoxic effects on retinal pigment epithelial and corneal cell cultures exposed to light radiation having a wavelength centred at 405 nm and to compare the experimental results with the most recent scientific literature and with the thresholds for photobiological risk provided by current standards.

## 2. Results

### 2.1. VBL Radiation Does Not Affect Ocular Cell Survival

To investigate the effects of VBL radiation on ocular cell survival, ARPE-19 (retinal pigmented epithelial cells) and BCE C/D-1b (endothelial cells from bovine cornea) were exposed to light radiation, and cell viability was assessed using an MTT assay.

**Figure 1:**
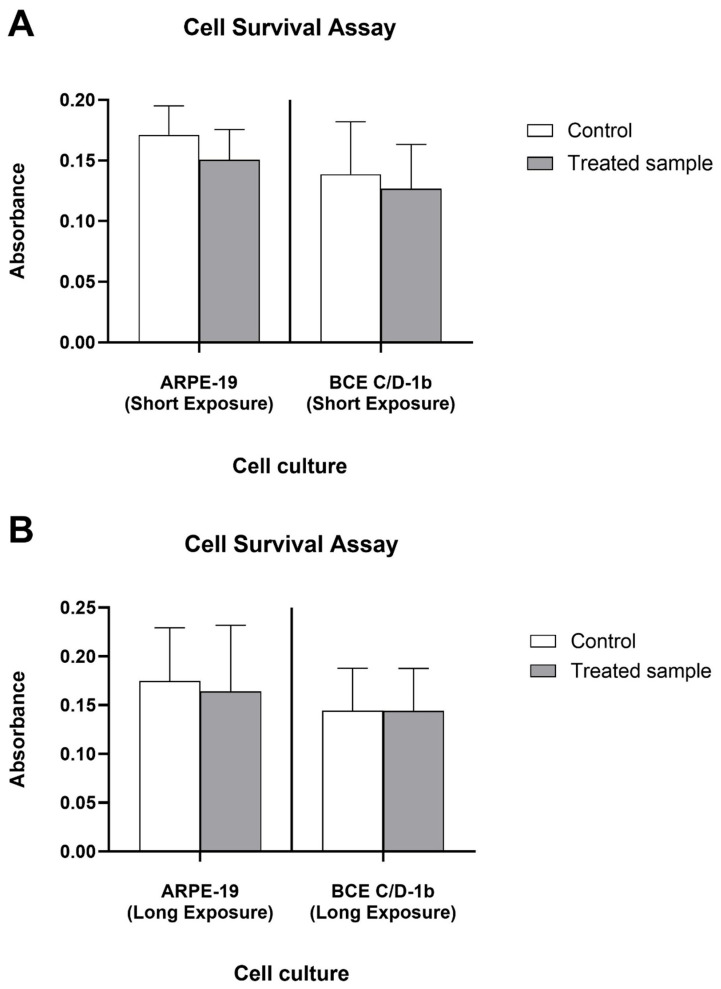
Cell Survival Assay Results. The bar graph shows absorbance values for ARPE-19 and BCE C/D-1b cell cultures under control and treated conditions for both short (3 h) (**A**) and long exposure times (9 h); (**B**). Absorbance is measured as an indicator of cell survival, with higher values indicating greater cell viability. Data are expressed as mean ± standard deviation of *n* = 3 experiments (**A**), and *n* = 7 experiments (**B**).

To mimic the potential exposure experienced by indoor workers during professional activities, a brief exposure was performed. After 3 h of exposure, ARPE-19 cultures showed a reduction in cell number of approximately 13.2% (0.061 log_10_ reduction, CI 0.05–0.07) compared to the unexposed control (Figure 1A). Similarly, BCE C/D-1b cells showed a reduction in cell survival with a value of 12.3% (reduction of 0.057 log_10_, CI 0.03–0.08) (Figure 1A). Subsequently, to evaluate the effect of prolonged exposure to light radiation, ocular tissue cells were exposed to light radiation for 9 h. Under these experimental conditions, a reduction in cell survival of ARPE-19 by 12.7% (0.06 log_10_ reduction, CI 0.04–0.08) was observed (Figure 1B). Finally, BCE C/D-1b cells treated for 9 h showed a reduction in cell viability of approximately 1.4% (reduction of 0.008 log_10_, CI 0.00–0.02) (Figure 1B).

**Figure 2:**
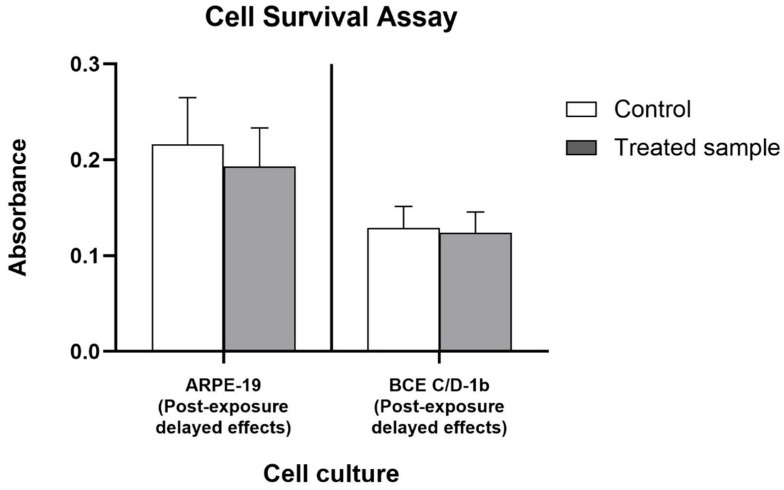
Delayed effects on cell survival after exposure. The bar graph shows absorbance values for ARPE-19 and BCE C/D-1b cell cultures under control and treated conditions, indicating delayed effects after exposure. Absorbance measures cell viability, with higher values representing greater cell survival. Data are expressed as mean ± standard deviation of *n* = 3 experiments.

To assess the late effects of light radiation on the cellular survival of ocular tissue cells, a three-hour light exposure was followed by an incubation in 5% FBS for 18 h. The results showed a reduction in cell survival of ARPE-19 by 12.6% (0.06 log_10_ reduction, CI 0.04–0.07) (Figure 2). Like previous results, cultures of BCE C/D-1b cells, exposed and subsequently treated to establish delayed effects, showed almost no reduction compared to controls (Figure 2).

From the collected data, we can conclude that blue light radiation exerted a weak decrease in cell viability on ocular tissue cells after both short and long exposures.

### 2.2. VBL Radiation Does Not Induce Oxidative Stress in Ocular Cells

Light radiation exposure, in particular UV, is one of the most important environmental sources of oxidative stress, which can damage proteins, lipids and DNA [37]. To assess the levels of oxidative stress induced by light radiation, we investigated its consequences on ROS production.

**Figure 3:**
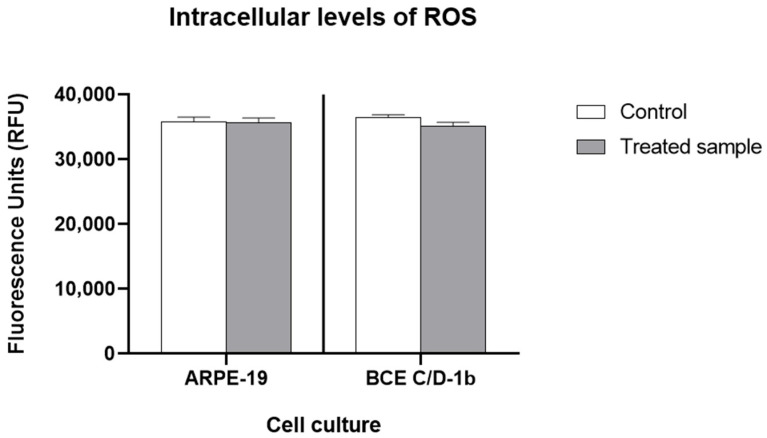
Intracellular ROS levels in ARPE-19 and BCE C/D-1b cell lines. The graph shows fluorescence units (RFU) indicating ROS levels in control and treated samples for both cell cultures after 3 h of light exposure. No significant difference in ROS levels is observed between control and treated samples within each cell line. Data are expressed as mean ± standard deviation of *n* = 4 experiments.

The results showed that in both cell lines, no biologically significant differences between the control and the irradiated samples were observed, and again the ARPE-19 cell line was slightly more sensitive than the corneal cell culture. ARPE-19 cells showed a decrease in ROS production of 1.82% (0.008 log_10_, CI 0.00–0.01) compared to the control, while BCE C/D-1b cells showed a decrease of 4.38% (0.019 log_10_, CI 0.00–0.01) (Figure 3).

**Figure 4:**
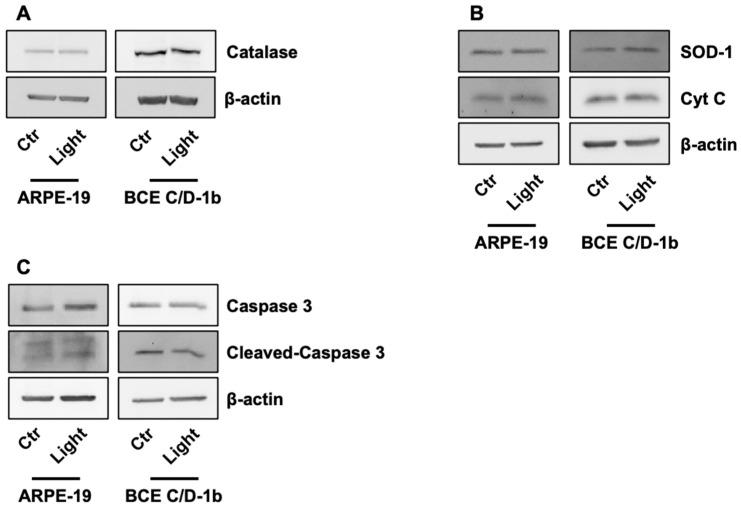
Effect of light irradiation on the expression of enzymes involved in oxidative stress and apoptosis. Expression of catalase (**A**), SOD-1 and Cyt C (**B**) evaluated by Western blot in ARPE-19 and BCE C/D-1b after 5 h of irradiation. (**C**) Activation of caspase-3 assessed by cleavage using Western blot analysis in ARPE-19 and BCE C/D-1b cells after 5 h of light exposure. β-actin was used as loading control. Blots are representative of three independent experiments.

To validate the ROS production data, we evaluated the expression of key enzymes involved in redox regulation, namely the antioxidant enzymes catalase and superoxide dismutase 1 (SOD-1). No changes in catalase and SOD-1 expression were observed after 5 h of light exposure in both cell lines (Figure 4A,B). It is noteworthy that BCE corneal cells inherently express higher levels of catalase, presumably to detoxify ROS resulting from physiological light exposure of the anterior tissue.

It has been reported that blue light can induce apoptosis in ocular cells [38]. To assess the initiation of apoptosis in our model, we examined the levels of cleaved caspase-3, the final effector of programmed cell death. In our cells, exposure to light radiation did not promote the activation of cleaved caspase-3, and no changes were observed in the total form of the protein (Figure 4C). Consistently, cytochrome c levels did not show any increase or detectable accumulation in either cell line, further indicating that mitochondrial apoptotic signalling was not activated under our experimental conditions (Figure 4B).

## 3. Discussion

The application of VBL within the 405–420 nm wavelength range for environmental disinfection has gained attention due to its antimicrobial properties [39]. VBL at 405–420 nm exerts its antimicrobial effects primarily through the generation of ROS [40]. When microbial chromophores absorb VBL, ROS are produced, causing oxidative damage to cellular components such as DNA, proteins, and lipids, ultimately leading to cell death. This photodynamic inactivation mechanism has been demonstrated to be effective against a variety of pathogens, including bacteria and fungi.

Despite these uses, current evidence underscores potential risks to eye health, circadian rhythms, and skin integrity. Prolonged exposure to VBL has been associated with retinal damage and photochemical injury. This can potentially lead to conditions such as age-related macular degeneration (AMD). Several studies suggest that the retina’s photoreceptor cells can undergo oxidative stress and apoptosis when exposed to high-intensity VBL, underscoring the need for protective measures such as VBL filters in screens and eyewear [41]. Further, VBL influences the suppression of melatonin, a hormone crucial for regulating sleep–wake cycles [42]. VBL can penetrate the skin and generate reactive oxygen species (ROS), leading to oxidative stress. This stress can result in inflammation, premature skin ageing, and potentially exacerbate conditions such as hyperpigmentation [43].

In this study, we assessed the ocular safety of VBL by using two cell types from different compartments of the eye by employing corneal endothelial cells (BCE C/D-1b) and retinal pigmented epithelial cells (ARPE-19).

Our study demonstrates that the exposure of corneal and retinal (i.e., pigment epithelial) cells to VBL is not harmful in terms of cell survival, ROS production, and induction of the apoptotic process. We assessed the effect of light radiation in two types of stimulation setting: short and long-time exposure, based on the total dose received [44]. We found that after 3 h of light irradiation (short exposure), VBL induced a marginal decrease in cell viability in the cells tested (corneal and retinal). Similarly, to assess the late effects of light radiation, after being exposed to light radiation for 3 h, the cells were cultured for an additional 18 h to determine if the exposure impacted their proliferative capacity. No significant effect on viability was found. Nevertheless, significant damage occurs when prolonged exposure to light irradiation affects photoreceptors activated by specific wavelengths, especially rhodopsin, which impacts these photoreceptors [45]. When we tested the effects of prolonged light exposure (9 h) on cells, slight effects were found.

The heightened production of ROS triggers oxidative stress, autophagy, inflammation and cell death [46]. On this basis, we assessed ROS production in ocular cells exposed to light; no burst of ROS was measured after 3 h of exposure to VBL. No increases in ROS or imbalances in the expression of key enzymes involved in maintaining the cellular redox state (i.e., catalase, SOD-1) were observed. In BCE corneal cells, catalase expressions is higher compared to ARPE cells, which are of retinal origin. This can be attributed to the cornea’s more external location, making it more exposed to light-induced ROS and spontaneously more prone to its self-protection.

According to the above results, no activation of apoptosis was found in either cell line.

Overall, these studies provide a theoretical basis for the safety of VBL in ocular tissues.

Our findings provide preliminary mechanistic evidence of the cellular effects of near-UVA radiation in a controlled environment. While these data are not sufficient to inform regulatory revisions, they may contribute to the broader scientific discussion on photobiological risk assessment. Current standards, such as IEC 62471-7:2023 [47], govern the safe use of non-coherent optical radiation. Considering its potential hygienic applications in occupied settings, further evaluation within the current regulatory framework may be warranted to ensure both effective implementation and continued protection of human health. 

Study limitations: Although our study provides valuable baseline data using a highly effective and standardized 3D-printed irradiation setup, several biological limitations must be acknowledged. First, the in vitro models employed, such as retinal pigment epithelial (ARPE-19) and bovine corneal (BCE C/D-1b) cells, exhibit significant metabolic resilience and do not capture the full physiological complexity and vulnerability of the human eye. Specifically, our model lacks photoreceptors and the broader neuroretinal network, which are known to be highly sensitive to light-induced damage. Second, and most critically, standard ARPE-19 cultures do not contain lipofuscin (LG) or melanin granules, which are naturally present in human tissue. In vivo, particularly in the ageing eye, the accumulation of LG in the RPE acts as a photoinducible free radical generator [48,49,50]. The presence of LG significantly increases the susceptibility of RPE cells to phototoxicity, oxidative stress, and apoptosis when exposed to violet-blue light [51,52]. Therefore, the relatively high cytocompatibility and the weak decrease in cell viability observed in our standard ARPE-19 model may not fully reflect the physiological risks to human RPE. Consequently, our findings should be interpreted as preliminary cytocompatibility screening. Future studies employing ARPE-19 cells loaded with LG, or more complex organotypic retinal models, are necessary to fully characterize the ocular safety profile of 405 nm light. Furthermore, given these biological caveats and the potential for long-term cumulative effects, the use of protective eyewear with yellow filters (blocking wavelengths up to 450 nm) should be considered as a standard precautionary measure for operators during light-based disinfection procedures in medical practice.

## 4. Material and Methods

### 4.1. Experimental Setting

**Figure 5:**
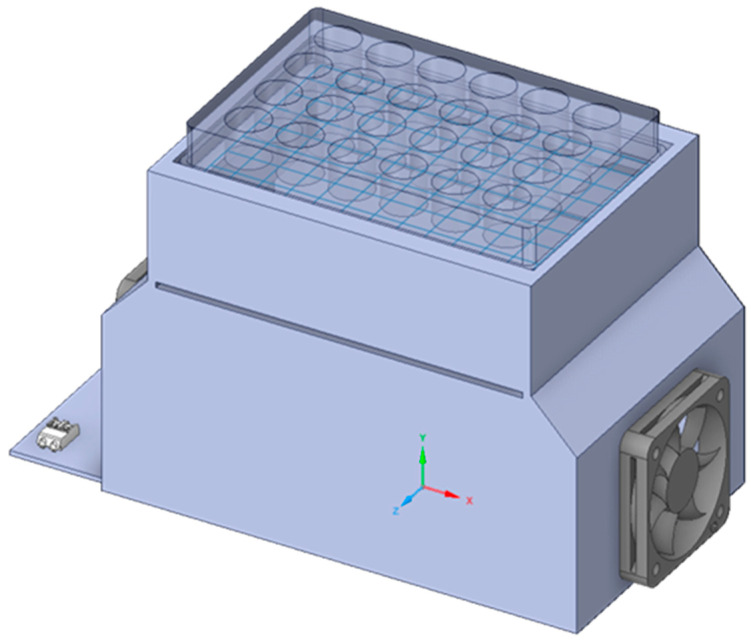
3D representation of the illumination device used for testing. The 405 nm light, emitted from the LED at bottom of the device, irradiates the cell cultures in the multiwell plate from bottom to top.

The light source utilized comprises a cluster of 14 LEDs (SST-10-UV-A130-F405-00, Luminus Devices, Sunnyvale, CA, USA) with a nominal peak wavelength of 405 nm. According to the manufacturer’s specifications, the LEDs exhibit a peak wavelength bin of 405–410 nm and a full width at half maximum (FWHM) spectral bandwidth of approximately 10 nm at 500 mA drive current. The viewing angle of the LED is 130°. The wavelength reported throughout the manuscript refers to the nominal peak emission. To prevent the LEDs from overheating excessively and deteriorating, as well as to avoid excessive heat affecting the cell culture during experiments, the source was connected to an aluminum heat sink at its base. Positioned above the light source is a rectangular black polylactic acid (PLA) stand measuring 17 cm × 10 cm × 12 cm, which supports the multiwell plate and directs the light radiation (Figure 5).

This stand was designed using CAD Solidworks 2020 software (Dassault Systèmes, Waltham, MA, USA) and made with a 3D printer (Ultimaker S5 B.V., Utrecht, The Netherlands). A liquid dissipation system was mounted under the PCB to limit the overheating of the LEDs inside the incubator (thus preventing their degradation), in addition, a ventilation system made of two fans was installed on the two smaller opposite walls of the stand to facilitate ventilation and to limit temperature excursion produced by the LEDs on the cell cultures during the period of exposure to the 405 nm light. The temperature of the LEDs and the multiwell plate was monitored with temperature probes throughout the experiments.

**Figure 6:**
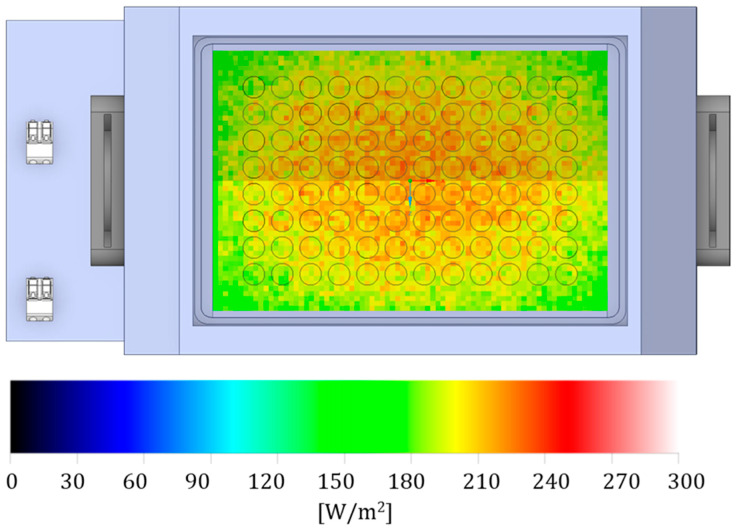
Photoradiometric simulation [W/m^2^], produced using Ansys Speos software (Ansys Inc., Canonsburg, PA, USA). The image shows light distribution in the different wells of the multi-well plate. Light scattering has been optimized to ensure efficient light distribution across the wells.

Electromagnetic spectrum characterization was carried out using an Avantes ULS2048CL EVO spectrophotometer (Avantes, Apeldoorn, Netherlands). The LED emission peak occurs at a wavelength of 405 nm. The energy map of the multiwell plate was measured to map light irradiance distribution (Figure 6).

### 4.2. Simulation Model

The setup for testing, the number of LEDs and placement in the device used for the tests was created using Ansys Speos software. A simulation was conducted to ensure even light distribution in all plate wells and measure light distribution at 405 nm.

### 4.3. Cell Culture

Bovine corneal endothelial cells (BCE C/D-1b) were purchased from IZSLER (Istituto Zooprofilattico Sperimentale della Lombardia e dell’Emilia-Romagna, Brescia, Italy). They were grown in an endothelial growth medium (EGM-2) containing a vascular endothelial growth factor (VEGF), recombinant human long R3 insulin-like growth factor 1 (R3-IGF-1), human epidermal growth factor (hEGF), human fibroblast growth factor (hFGF), hydrocortisone, ascorbic acid, heparin and GA-1000 (Lonza Group, Ltd., Basilea, Switzerland), 2 mM glutamine, 100 units/mL penicillin, 100 µg/mL streptomycin (Merck KGaA, Darmstadt, Germany) and 10% fetal bovine serum (FBS) (Cytiva/HyClone, Wilmington, DE, USA).

ARPE-19 human normal pigmented epithelial cells were provided by IZSLER. They were maintained in DMEM F-12 (Lonza Group, Ltd., Basilea, Switzerland). The medium was supplemented with 2 mM L-glutamine, 100 units/mL penicillin, 100 µg/mL streptomycin and 10% FBS (Cytiva/HyClone, Wilmington, DE, USA). The cells were grown at +37 °C in an incubator with 5% CO_2_.

### 4.4. Survival Assay

Cell survival was quantified by an MTT assay [53]. Light radiation was performed following three protocols: (1) short exposure for 3 h in serum-free medium; (2) 9 h of light radiation in medium with 2% FBS to mimic prolonged exposure and (3) treatment for 3 h followed by 18 h of culture in medium with 5% FBS to establish delayed effects. The energy dose delivered to the cell cultures was approximately 21 mW/cm^2^. Due to the different exposure times between the different protocols, the light output of the device was adjusted for each protocol to keep the energy dose administered constant. Control cells for each protocol were kept in an incubator without radiation, at the same medium conditions as the treated ones.

For all, 3 × 10^3^ cells/well were seeded in 96-multiwell plates in a medium with 10% FBS. After adherence, the medium was replaced with a serum-free medium, and the cells were exposed to light radiation. At the end of the stimulation, the medium was removed, and cells were incubated for 4 h with a fresh medium in the presence of 1.2 mM 3-(4,5-dimethylthiazol-2-yl)-2,5-diphenyltetrazolium bromide (MTT) (Merck KGaA, Darmstadt, Germany). After solubilization of formazan crystals with DMSO, absorbance was measured with a microplate absorbance reader (Infinite 200 Pro, Tecan Life Sciences, Männedorf, Switzerland) at 540 nm. Absorbance data are reported as overall averages of treated samples and positive controls, along with 95% confidence intervals, and comparisons between the two groups are reported as percentage reduction.

### 4.5. ROS Measurement

ROS levels were evaluated as previously reported [54]. A total of 3 × 10^3^ cells/well were seeded in a 96-multiwell plate and, after adherence, were irradiated for 3 h in a medium without a serum. DCFH2-DA (2,7-dichlorodihydrofluorescein diacetate; Invitrogen, Italy) was added (10 μm, 30 min) and intracellular levels of ROS were evaluated with a microplate reader (excitation/emission 495/527; Infinite 200 Pro, Tecan Life Sciences, Männedorf, Switzerland). The results are reported as relative fluorescence units (RFU) corrected for the cell number counted/well.

### 4.6. Western Blot

Cells (2 × 10^5^/well in 6 multiplate) were exposed to light irradiation for 5 h, a time consistent with caspase-3 activation [55]. The expressions of markers of apoptosis and the antioxidant enzyme catalase and Superoxide Dismutase-1 (SOD-1) were evaluated by Western blot as previously described [56].

Anti-caspase-3 (rabbit, 1:1000, cat. n. 9662), anti-cleaved caspase-3 (Asp175) (rabbit, 1:1000, n. 9664) and anti- Cytochrome C (Cyt C) (rabbit, 1:1000 cat. n 11940) were provided by Cell Signaling Technology, Inc. (Danvers, MA, USA). Anti-catalase (mouse, 1:1000 cat. n. C0979); anti SOD-1 (rabbit, 1:1000 cat. n. SAB5701040) and anti-β-actin antibody (mouse, 1:10,000, cat. no. MABT825) were from Merck KGaA (Darmstadt, Germany).

### 4.7. Statistical Analysis

Results are representative of the average of at least 3 independent experiments. The absorbance data of the exposed cultures in the different wells of the plate were corrected according to the weighted average of the irradiance emitted by the light source. This step minimized the variable light energy, which was not perfectly uniform in all the wells used. Statistical analysis was conducted using STATA 17 software (StataCorp LLC, College Station, TX, USA). Absorbance values were analyzed as continuous outcomes. For each experiment, exposed samples were compared to the corresponding non-exposed controls by calculating the log-transformed ratio:

$${>\Delta }_{i}=\log_{10}\left(\frac{C}{E_{i}}\right),>$$

where C is the mean control value and $E_{i}$ is the value for the exposed replicate. The mean $\Delta_{i}$ and its 95% confidence interval were estimated using the standard error (SD/√n) and were back transformed to express the percent change relative to controls.

## Data Availability

The datasets used and/or analyzed during the current study are available from the corresponding author on reasonable request.
